# Standardized Testing for Thermal Evaluation of Bone Drilling: Towards Predictive Assessment of Thermal Trauma

**DOI:** 10.3390/bioengineering11070642

**Published:** 2024-06-24

**Authors:** Sihana Rugova, Marcus Abboud

**Affiliations:** 1 Department of Oral Biology and Pathology, Stony Brook University, Stony Brook, NY 11794, USA; hanarugova@gmail.com; 2 School of Engineering, Stony Brook University, Stony Brook, NY 11794, USA

**Keywords:** bone drilling, infrared thermography, orthopedic surgery, implant dentistry, heat, osteotomy, bone cutting, osteocytes

## Abstract

To ensure the prevention of thermal trauma and tissue necrosis during bone drilling in surgical procedures, it is crucial to maintain temperatures below the time- and temperature-dependent threshold of 50 °C for 30 s. However, the absence of a current standard for assessing temperatures attained during bone drilling poses a challenge when comparing findings across different studies. This article aims to address this issue by introducing a standardized testing method for acquiring thermal data during experimental bone drilling. The method requires the use of three controlled variables: infrared thermography, standard bone blocks, and a regulated drilling procedure involving a drill press with irrigation that simulates a surgeon. By utilizing this setup, we can obtain temperature data that can be effectively applied in the evaluation of other variables, such as surgical techniques or drill bit design, and translate the data into bone damage/clinical outcomes. Two surgical drill bits (2.0 mm-diameter twist drill bit and 3.3 mm-diameter multi-step drill bit) are compared using this experimental protocol. The results show the 2.0 mm bit reached significantly higher temperatures compared to the 3.3 mm bit when preparing an osteotomy (*p* < 0.05). The 2.0 mm drill bit reached temperatures over 100 °C while the 3.3 mm drill bit did not exceed 50 °C.

## 1. Introduction

Drilling into or through bone is a routine surgical procedure as bones protect vital structures and provide support and structure for muscles. Among the 14 surgical specialties recognized by the American College of Surgeons, more than half regularly incorporate some form of bone-drilling or cutting procedure during surgery [1]. It is crucial that these procedures are carried out with minimal trauma. Even in cases where the surgery does not directly focus on a bone-related procedure, i.e., the fixation of tissue to bone with screws, the potential of complications resulting from excessive heat generation during bone drilling or cutting can have significant clinical ramifications, leading to detrimental clinical complications and an unsuccessful surgery [2,3,4,5,6].

Assessing the temperatures reached during bone surgery provides a means to quantify the trauma incurred. The temperature at which bone necrosis begins is directly influenced by two factors: time and temperature. In our experiments, we use a threshold of 50 °C for 30 s to indicate irreversible bone damage [7]. As the temperature surpasses this threshold, damage to surrounding tissue worsens. As the duration of heat exposure lengthens, the radius of damaged tissue increases, often affecting blood flow first. Collagen denatures at 60 °C, and bone alkaline phosphatase at 56 °C or higher when the exposure time is 10 s or longer. Diaphorase, an enzyme essential for cellular aerobic and anaerobic functions, begins to deactivate at 50 °C. Irreversible changes to dog femoral bone tissue in vivo were seen at 50 °C, in rabbit tissue at 47 °C for one minute, and in Lundskog’s study, at 50 °C for 30 s. Immediate cell death occurs at 70 °C, with tissue death extending further as the duration of heat exposure to living tissue lengthens [7,8,9,10,11,12,13].

Understanding the risks associated with excessive heat on bone is vital for comprehending the trauma that may result from surgeries. Researchers have developed various techniques to measure temperatures during bone-drilling or cutting procedures [14]. However, due to a lack of standardization, comparing studies has proven challenging. In this article, we establish a protocol for measuring the heat generated during experimental bone drilling or cutting that enables the evaluation of specific variables of interest and their impact on heat production during surgical bone-drilling or cutting procedures.

## 2. Materials and Methods

A custom-built drill press was used to ensure a standard, unbiased drilling procedure for each osteotomy. Ten mm-deep osteotomies were drilled into standard bovine blocks specifically prepared for testing. External irrigation was used throughout, and an infrared camera acquired temperature data during the drilling procedure.

Bone Specimens: Artificial bone similes (BS180035-120035-180035, BoneSim, Cassopolis, MI, USA) with a 3 mm cortical layer, 12 mm cancellous layer, and diameter of 58 mm were used as the medium in which the osteotomies were prepared. These engineered matrices of reconstituted viable bone represent type 2 bone as described by Lekholm and Zarb. The organic bone samples have properties similar to human cortical bone, as shown in Table 1 [15,16].

**Figure 1:**
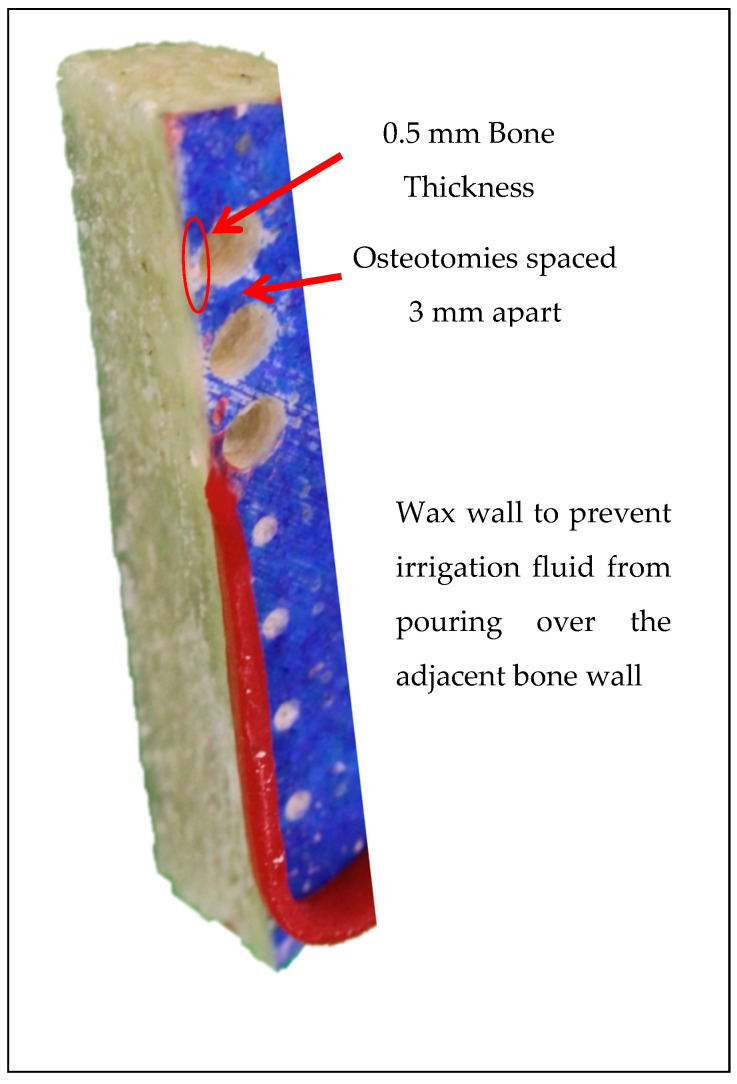
CNC-machined bonesims.

Using a high-precision Computer Numeric Control (CNC) machine, the bonesims were cut into strips with a 10 mm width. Each osteotomy site was marked so that its periphery, post-osteotomy, was at least 3 mm away from the next osteotomy site as well as exactly 0.5 mm away from the lateral bone wall that the infrared camera faces, allowing the infrared camera to measure the temperatures reached at 0.5 mm away from the osteotomy (Figure 1). The bonesim strips were soaked in room temperature saline for at least 20 min prior to drilling to simulate a more fluid environment.

**Figure 2:**
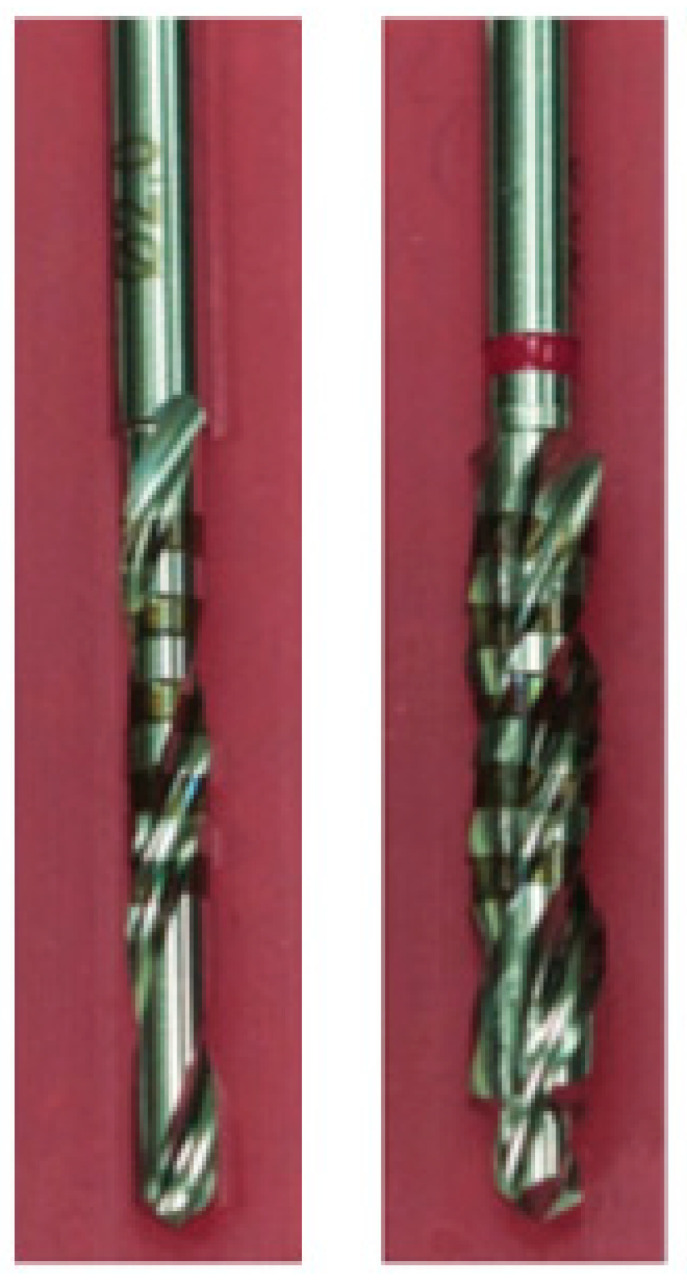
Image of drill bits tested: ø2.0 mm (**left**); ø3.3 mm (**right**).

Drills and Drilling Groups: Two different surgical drills bits for osteotomy preparation were compared using the standardized testing set up (Table 2). The first surgical drill bit was a straight/parallel wall shaped 2.0 mm-diameter twist drill. The second surgical drill bit started with a diameter of 2.0 mm, then a step after 2 mm with an increase in diameter to 3.3 mm; the remainder of the drill bit had a straight/parallel shape. Both were externally irrigated at a flow rate of approximately 12 mL/min. All drill bits used were manufactured from surgical steel 1.4197 in Germany. See Figure 2 for images of drill bits tested. Each drilling procedure was performed independently, so no sequential drilling was performed.

Surgical drill bits were used to prepare an osteotomy using the custom-built drill press with a load of 2.3 kg in combination with a W&H implant motor (W&H Group, Bürmoos, Austria) set to a spindle speed of 1500 rpm. Each new surgical drill bit was used ten times under these testing conditions. The motor and spindle speeds chosen are within the range of those used in implant surgery for the placement of an endosseous dental implant.

Statistical Analysis: The data analysis for this study was generated using the Real Statistics Resource Pack software (Release 3.8); copyright (2013–2015) Charles Zaiontz; www.real-statistics.com. The temperatures achieved under each condition were recorded and compared statistically by a student’s *t*-test. Statistical significance was established as *p* < 0.05; n = 10.

Temperature device: One FLIR A325sc infrared camera (FLIR Systems Inc., Wilsonville, OR, USA) fixed with a close-up 4× lens was positioned on a tripod 80 mm from the edge of the bonesim, separated from the final osteotomy by 0.5 mm of bone wall. The infrared camera had a standard temperature range of 0–152.1 °C and accuracy of ±2 °C or ±2% of its reading. A wax barrier at least 5 mm-high was built on the outer top edge of the bonesim to prevent any irrigation water from pouring onto the outer lateral surface of the bonesim, obstructing the view of the infrared camera. Videos of the osteotomy procedure were recorded on the lateral surface of the bonesim in a dynamic rainbow palette (20 colors) using FLIR ResearchIR Max version 1 on Windows 8.1. Temperature readings were recorded continuously before, during, and after the osteotomy procedure to determine the maximum bone temperatures (in Celsius) and duration (in seconds) of temperature influence. Base temperatures were normalized to 32 °C to match the maximum maxillary temperature recorded in literature [17]. Tissue damage is both time- and temperature-dependent. The threshold for irreversible tissue damage in this study was 50 °C for 30 s, meaning anytime the 50 °C temperature threshold was exceeded, the duration the osteotomy remained above the temperature threshold was recorded [18]. Temperatures between 50 °C and 70 °C indicate bone cell damage. If the temperature remains in the range of 50–70 °C for 30 s or longer, then the damage produced is irreversible. Temperatures at or above 70 °C indicate immediate osteocyte death regardless of duration.

**Figure 3:**
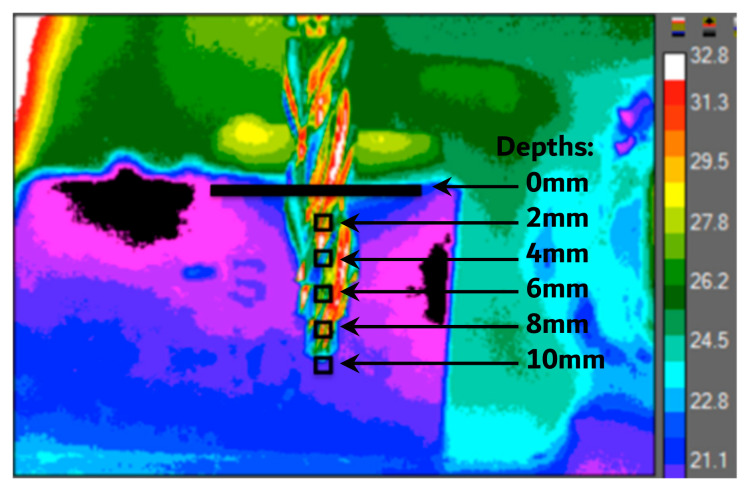
A still image of a surgical drill bit placed in front of a bonesim at a depth of 10 mm is shown. All drilling procedures began with such images for proper orientation prior to video analysis. The temperature data are collected within the black boxes throughout the osteotomy procedure at intervals of 2 mm depths directly centered in front of the osteotomy.

Thermal video recordings were analyzed to document the maximum temperature readings at 5 regions of interest, at depths of 0, 2, 4, 6, 8, and 10 mm, 0.5 mm away from the periphery of the osteotomy throughout the duration of the procedure (Figure 3).

## 3. Results

**Figure 4:**
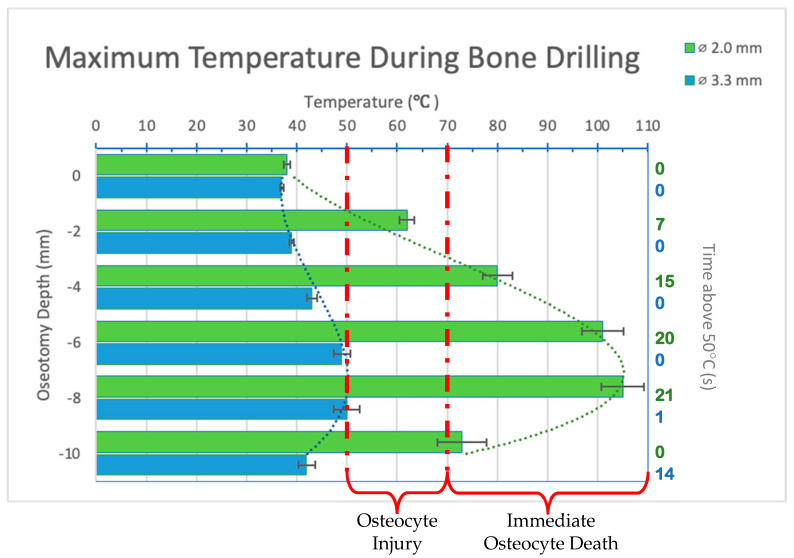
Bar graph showing maximum temperatures reached when drilling into bone to a depth of 10 mm. Temperature range of bone cell injury and bone cell death are marked on graph. Left axis shows drilling depth while top axis shows maximum temperature reached at that depth. Right axis shows duration (in seconds) temperatures exceeded 50 °C. Green color refers to 2.0 mm-diameter drill bit (ø2.0 mm). Blue color refers to 3.3 mm-diameter drill bit (ø3.3 mm). Temperatures are normalized to maxillary temperature (32 °C).

Infrared videos taken during the osteotomy procedure were analyzed, and maximum temperatures at depths of 0, 2, 4, 6, 8, and 10 mm were recorded and organized into a bar graph (Figure 4). For the 2.0 mm-diameter drill bit at a 2 mm depth, reversible bone cell damage was expected as the maximum temperature reached was 62 °C, exceeding the first temperature threshold of 50 °C and lasting for 7 s. At depths of 4, 6, 8, and 10 mm, temperatures exceeded the 70 °C threshold, indicating immediate cell death and irreversible tissue damage independent of time. For the 3.3 mm-diameter drill bit, the temperatures reached never exceeded 50 °C for 30 s. At a drilling depth of 8 mm, 50 °C was reached for 1 s.

The maximum temperatures reached by the 2.0 mm-diameter drill bit were statistically significantly higher than those achieved with the 3.3 mm-diameter drill bit at all drilling depths (*p* < 0.05). Peak temperatures were reached at 8 mm for both drill bits. The longest duration of heat exceeding 50 °C was also at the 8 mm depth for both drill bits.

**Figure 5:**
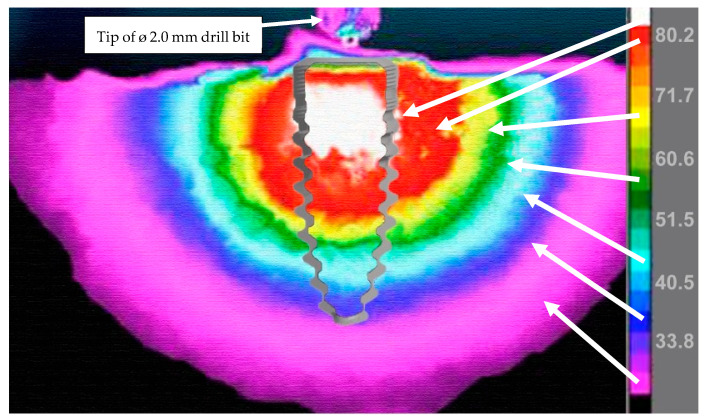
Still image of a recording with infrared camera showing heat spreading through the osteotomy immediately after bone drilling with the 2.0 mm drill bit is completed. The tip of the drill bit is shown at the top of the image. The temperature scale (in Celsius) is shown on the left of the image using a rainbow palette temperature gradient. An outline of a 3.5 mm-diameter endosseous dental implant is shown overlaying the osteotomy site to visualize how heat would impact the implant site. Arrows match the temperature scale to the osteotomy to help identify which temperatures are reached and to visualize how far heat spreads beyond the osteotomy site.

**Figure 6:**
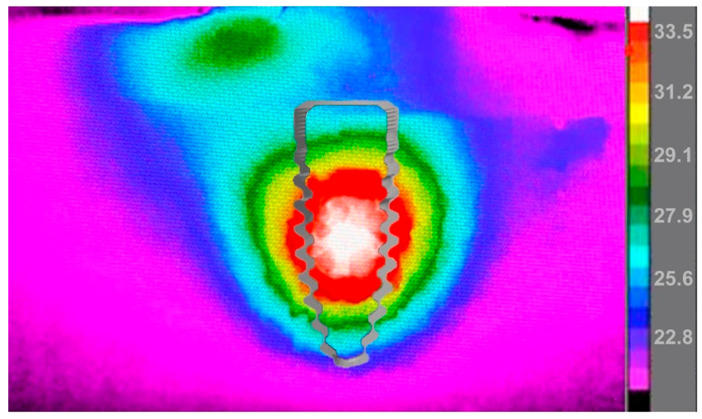
Still image of a recording with an infrared camera showing heat spreading through the osteotomy immediately after bone drilling with the 3.3 mm drill bit is completed. The temperature scale (in Celsius) is shown on the left of the image using a rainbow palette temperature gradient. An outline of a 3.5 mm-diameter endosseous dental implant is shown overlaying the osteotomy site to visualize how heat would impact the implant site.

The still image of the infrared recording for the 2.0 mm drill bit (Figure 5) shows that even when drilling was complete, the surrounding bone needed time to dissipate the high temperatures reached. Temperatures remained high immediately at the implant site and continued to be over 50 °C 2 mm away from the osteotomy. The 3.3 mm drill bit (Figure 6) did not reach temperatures over 50 °C during the bone-drilling procedure, and therefore did not exceed 50 °C when the osteotomy was completed.

## 4. Discussion

During surgical procedures involving bone drilling or cutting, the generation of heat is a critical concern as it can lead to varying degrees of tissue damage. The trauma endured ranges from transient cellular effects to irreversible harm depending on the temperature reached and exposure time. Our investigation into two different drill bit sizes revealed significant differences in the temperatures produced when drilling to a depth of 10 mm, concluding that the 2.0 mm twist drill bit produces higher temperatures compared to the wider drill bit diameter of 3.3 mm. This variance likely stems from distinct design features between the drill bits, including flute angle, cutting edges, and flute volume, all of which influence the removal of bone debris from the surgical site [19].

Infrared video observations underscore the thermal anisotropic nature of bone, highlighting how heat generated during drilling remains localized and dissipates over a short distance [20,21]. This has implications across various surgical disciplines, from dentistry where adjacent teeth are at risk, to orthopedics and implant surgery where complications such as bone sequestration may arise from thermal trauma. While immediate complications can often be managed with antibiotics and pain medications, long-term effects manifest through vascular changes and alterations in bone structure. Capillary systems diminish and osteocytes are replaced by adipocytes [13,22,23].

In articles related to thermal trauma in bone, 47 °C for 1 min is often used as the threshold for damage to osteocytes [24]. However, we chose an earlier article that uses 50 °C for 30 s because we found that 1 min is an excessive duration for bone-drilling or cutting procedures. In our observations, if the temperature exceeds 47 °C for 1 min during a bone-drilling procedure, due to the anisotropic property of bone, the temperature has likely already surpassed 70 °C. Additionally, in implant dentistry, it is extremely uncommon for a surgeon to drill continuously in the same spot for 1 min, making this time threshold less practical for clinical applications.

By analyzing temperature data during osteotomy preparation, we can better understand how our instruments and surgical techniques thermally impact bone. Some variables we can evaluate include tool design, tool rotations or oscillations per minute, feed rates or applied pressure by the doctor, tool diameter, tool material or coating, surgical techniques such as sequential drilling, one-drill protocols or guided surgery, and bone density. This insight not only aids in predicting surgical outcomes but also guides adjustments to treatment plans and surgical techniques. Contrary to conventional wisdom, our findings challenge the notion that sequential drilling can mitigate heat; it may actually exacerbate thermal trauma, particularly when starting with smaller-diameter drill bits such as the 2.0 mm-diameter twist drill used in this study and in most dental implant procedures.

In previous experiments, we compared an infrared device to a thermocouple for collecting temperature data and found that the infrared camera provides higher-quality and more diverse data [19]. The visualization of heat alone allows us to better understand the impact of our surgeries on patient outcomes, enabling us to make informed changes to our surgical protocols. Although the experimental design presented here is our preferred method for thermal data collection, it still presents challenges. Maintaining a 0.5 mm drilling distance from the lateral wall of the bone is difficult to achieve without a CNC machine to mark the drilling locations. Even when the 0.5 mm distance is maintained, irrigation fluid can still seep through the bone due to its porosity, rendering that data point unusable and requiring a retest as the infrared device can only measure the temperature of whatever is immediately in front of it. Despite these issues, we chose this method over thermocouples because thermocouples have limited direct contact with bone due to porosity, are cumbersome, and require multiple units to capture thermal data over a larger distance. Additionally, the biggest challenge with thermocouples is understanding the nature of the temperature data being collected. Discussions with manufacturers did not clarify whether the data represented maximum, average, or a combination of temperatures.

In presenting our research, we aim to establish a standardized protocol for measuring temperatures during bone-drilling and cutting procedures, with the ultimate objective of quantifying and assessing thermal trauma. This framework not only enhances our understanding of surgical limitations but also opens avenues for exploring alternative techniques or tools to minimize thermal risks during surgery.

## 5. Conclusions

This testing protocol enables the medical device industry, researchers, and clinicians to evaluate the heat generated by bone-cutting tools and develop solutions to reduce thermal trauma. Thermal trauma significantly impacts surgical outcomes by potentially causing bone damage, delayed healing, and scar tissue resulting in short- and long-term complications. By quantifying thermal trauma during bone-cutting and drilling procedures, researchers can establish a bone burn scale, leading to better techniques and tools that minimize heat-induced damage and improve patient recovery and surgical success rates.

## Figures and Tables

**Table 1:** Properties of human cortical bone compared to bonesims (Bonesim, Cassopolis, MI, USA).

Property	Human Cortical Bone	Bonesim
Hardness (Shore D)	85–95	90
Density (g/cc)	1.4–1.9	1.8
Comp. Strength (MPa)	100–182	110
Screw Insertion Torque (Nm)	1.36–1.58	1.47
Drilling Toughness (s/mm)	2.39	2.42
Thermal Conductivity (W/m/K)	0.3–12.8	0.3–0.4
Specific Heat (J/kg°C)	1260	1200–1300

**Table 2:** Diameters of surgical drill bits used to drill into bone similes.

Pilot ø2.0 mm twist drill bit
Multi-diameter drill bit: ø3.3 mm 1st diameter: 2.0 mm 2nd diameter: 3.3 mm

## Data Availability

Dataset available on request from the authors.
